# Dynamic Single‐Binding Event Profiling With on‐Chip Microlenses for Wash‐Free Digital Biosensing

**DOI:** 10.1002/advs.76076

**Published:** 2026-06-28

**Authors:** Tingting Zhan, Lianyu Lu, Guoqiang Gu, Pengcheng Zhang, Jienan Shen, Xiaotian Tan, Wei Ye, Shi Hu, Yi Zhang, Hao Yu, Shaoqin Liu, Hairong Zheng, Hui Yang

**Affiliations:** ^1^ Research Center For Biosensing and Intelligence Institute of Biomedical and Health Engineering Shenzhen Institutes of Advanced Technology Chinese Academy of Sciences Shenzhen China; ^2^ School of Medicine and Health Key Laboratory of Microsystems and Microstructures Manufacturing (Ministry of Education) Harbin Institute of Technology Harbin China; ^3^ State Key Laboratory of Biomedical Imaging Science and System Shenzhen Institutes of Advanced Technology Chinese Academy of Sciences Shenzhen China

**Keywords:** digital immunoassay, microlens chip, plasmonic nanoparticle, single‐binding event

## Abstract

Single‐molecule detection enables precise biomarker quantification, yet conventional endpoint assays suffer from long incubation and nonspecific binding. Kinetic assays resolve these via real‐time monitoring of molecular interactions, but their reliance on complex optics has restricted their practical adoption, especially in point‐of‐care settings where speed, portability, and ease of use are paramount. We present a microlens‐assisted platform that enables real‐time tracking of single‐binding events under conventional bright‐field microscopy. An on‐chip microlens array amplifies scattering from gold nanoparticle labels, enabling kinetic monitoring with low‐magnification optics. Dynamic fingerprints of individual events distinguish specific from nonspecific interactions, achieving wash‐free digital biosensing with high sensitivity and specificity. This approach not only preserves the analytical advantages of kinetic profiling but also enables an exceptionally compact and portable optical configuration. Using cardiac troponin I, a time‐critical biomarker for acute myocardial infarction, we demonstrate an ultralow detection limit of 0.051 pg mL^−1^ and validate clinical applicability in undiluted serum samples, paving the way for miniaturized, high‐performance biosensors that can deliver laboratory‐grade diagnostics at the point of need.

## Graphical abstract

A microlens‐enhanced platform enables real‐time tracking and digital counting of single‐binding events using conventional bright‐field microscopy. Amplified scattering signals from gold nanoparticle labels and kinetic signature analysis allow wash‐free discrimination between specific and nonspecific interactions, while providing kinetic and mechanistic insights inaccessible to ensemble‐averaged assays. This compact strategy demonstrates high sensitivity and clinical applicability, advancing miniaturized biosensors for single‐molecule‐resolved detection.

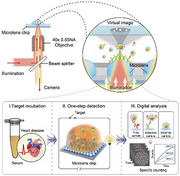

## 1 Introduction

Recent advances in single‐molecule detection have revolutionized biomarker quantification by transforming ensemble signals into discrete molecular events, enabling precise measurement of sub‐picomolar analyte concentrations. Traditional ensemble techniques, which rely on measuring bulk signals at reaction equilibrium, inherently lack the sensitivity to resolve rare events, as the weak response from collective target molecules is obscured within background noise [1, 2, 3]. In contrast, single‐molecule detection isolates and tracks individual binding events, capturing molecular heterogeneities and revealing subtle variations that are otherwise lost in ensemble measurements [4]. This capability significantly enhances the detection and quantification of rare biomarkers, pushing the limits of sensitivity beyond what conventional techniques can achieve [5].

However, the full understanding of biomolecular functions, particularly protein‐protein interactions, requires more than just counting static events. Many critical cellular processes are governed by the dynamics of these interactions, i.e., their association and dissociation kinetics, which embody a fundamental regulatory challenge. For instance, sensitive signal detection often necessitates high‐affinity binding, while rapid signal termination and environmental adaptability rely on fast exchange rates. Resolving these dynamic parameters is therefore crucial, yet it remains beyond the reach of conventional endpoint assays, which only provide a static snapshot after equilibrium is reached, a process that can take hours, especially for low‐abundance targets. This limitation is particularly acute in time‐sensitive clinical scenarios, such as the diagnosis of acute myocardial infarction (AMI), where the rapid and accurate quantification of cardiac troponin I (cTnI) directly impacts therapeutic decision‐making and patient survival. Yet endpoint assays are often constrained by diffusion‐limited binding and extended incubation, preventing simultaneous achievement of high sensitivity and rapid turnaround required in pre‐hospital or emergency settings.

Over the past decade, various single‐molecule detection technologies, such as Simoa, have demonstrated remarkable sensitivity, achieving limits of detection in the low‐femtomolar to attomolar range [6]. These approaches typically rely on spatially resolved strategies, utilizing microdroplet compartmentalization or microarrays to isolate and enumerate individual molecules with high precision [5, 7, 8]. However, these methods remain fundamentally endpoint in nature, requiring reactions to reach equilibrium before readout [9]. Consequently, diffusion‐driven mass transport limitations necessitate extended incubation times of several hours or even overnight for low‐concentration analytes [10, 11]. Furthermore, in complex biological matrices, single‐molecule detection faces additional challenges in maintaining both sensitivity and specificity. The binding affinity of probe molecules determines the fraction of targets successfully captured, leading to wide variability across different probe‐analyte pairs [12]. Even with rigorous washing steps, nonspecific interactions, which often involve extraneous molecules at significantly higher concentrations than the target, compromise measurement accuracy [13].

Kinetic assays have thus emerged as an effective alternative, enabling the extraction of detailed information about binding affinities, association and dissociation rates, and molecular interaction dynamics. This capability is particularly crucial at extremely low target concentrations, where distinguishing specific binding events from background noise becomes a major challenge [14]. By leveraging the unique kinetic signatures of binding events, kinetic assays offer unparalleled specificity, allowing rare biomolecular interactions to be identified with high confidence. However, their broader adoption has been hampered by reliance on complex, bulky optical systems, which limit their translation to compact, field‐ready devices. It is here that time‐resolved single‐molecule detection proves pivotal, as it offers a window into the dynamic nature of molecular interactions. This approach directly visualizes individual binding events through high‐resolution imaging. By tracking the dynamics of these isolated events, it not only captures molecular heterogeneities and subtle variations lost in ensemble measurements but also achieves the precise kinetic monitoring at the nanoscale that conventional methods struggle to deliver.

Recently, nanoparticle‐based probes have gained prominence as an alternative to fluorescent labels in kinetic biosensing due to their superior optical properties and resilience to photobleaching. While fluorescent probes are widely used in kinetic assays, their performance is fundamentally constrained by the finite photon budget of individual fluorophores, leading to limited observation times and reduced tracking precision. Additionally, single‐fluorophore detection requires complex optical setups such as total internal reflection fluorescence microscopy [15]. In contrast, light scattering from plasmonic nanoparticles, such as gold nanoparticles (AuNPs), provides a high‐contrast signal free from emission saturation or photobleaching, enabling stable and prolonged tracking of single‐molecule interactions [16]. Techniques such as dark‐field microscopy, surface plasmon resonance (SPR) microscopy, and interferometric microscopy are commonly employed for single‐nanoparticle visualization. However, these methods suffer from long acquisition times, reliance on high‐power illumination, and the requirement for high‐numerical aperture (NA) objectives, leading to sophisticated optical systems with limited field‐of‐view (FOV) and low temporal resolution. Since FOV dictates the number of nanoparticle probes that can be simultaneously monitored, small FOVs fundamentally restrict sensitivity and throughput.

An ideal optical system for dynamic single‐binding event detection must fulfill four key criteria: (i) direct observation of nanoparticle probes without additional processing steps (e.g., washing, complex image processing); (ii) a large FOV to accommodate more binding events; (iii) high temporal resolution for real‐time tracking; and (iv) a simplified optical path to facilitate practical applications, especially in compact and portable formats suitable for point‐of‐care use. A straightforward approach to expanding FOV is to employ low‐NA, low‐magnification objective lenses. However, this inherently compromises light collection efficiency, making it difficult to resolve and enumerate nanoparticles smaller than 100 nm.

To address this challenge, microscale dielectric microspheres, owing to their unique optical properties, have emerged as an efficient and cost‐effective solution for enhancing weak light collection in low‐magnification optical systems [17, 18, 19, 20]. These microspheres facilitate the visualization of nanostructures beyond the Rayleigh diffraction limit, significantly improving signal collection efficiency [21]. Microsphere‐assisted microscopy has been demonstrated in various configurations, including bright‐field [22], dark‐field, confocal [23], fluorescence [24], and interferometric microscopy [25]. While the exact theoretical mechanism behind microsphere‐assisted resolution enhancement remains a subject of debate, their great near‐field focusing and magnification capabilities have been successfully applied to diverse imaging modalities [26, 27]. Unlike conventional high‐NA setups, microsphere‐assisted optical systems offer a robust, scalable, cost‐effective, and user‐friendly solution for high‐resolution imaging of nanoscale objects such as plasmonic nanoparticles. This capability is especially critical in contexts such as AMI, where rapid and precise biomarker detection can significantly influence patient outcomes. However, despite their promising potential, microsphere‐assisted techniques have yet to be integrated on‐chip for real‐time single‐molecule kinetic assays under bright‐field microscopy in a truly portable format. The development of a compact, rapid, and accurate sensing platform could bridge the gap between symptom onset and clinical intervention, bringing laboratory‐grade diagnostics to the point of need, even into ambulances or other pre‐hospital settings.

In this work, we introduce a microlens‐based biosensing approach that enhances on‐chip light scattering for dynamically tracking and enumerating single‐binding events using a portable optical system. The system employs an incoherent light source and a low‐magnification objective in an inverted configuration. Crucially, it incorporates an on‐chip microlens array composed of high‐refractive‐index dielectric microspheres, which functions as a superlens layer to amplify the scattering intensity of AuNP probes. This enhancement enables rapid and efficient imaging of individual 80 nm AuNPs using a low‐magnification objective with white LED illumination. Unlike existing nanoparticle‐based optical sensing techniques, this approach facilitates direct localization and dynamic tracking of AuNP‐labeled binding events without requiring complex optics, making it uniquely suited for integration into field‐deployable devices. To validate our approach, we demonstrate its performance using cTnI, a gold‐standard biomarker for AMI that requires rapid and precise detection in clinical settings to prevent further cardiac damage. Operating under a miniaturized, cost‐effective optical system, the microlens‐assisted biosensing platform achieves ultrasensitive, high‐specificity detection at the single‐binding event level, delivering a wide dynamic range, high selectivity, and direct clinical applicability. Critically, by leveraging the kinetic signatures of individual binding events, the technique enables precise discrimination between specific and nonspecific interactions in a wash‐free manner. This unique capability, combined with the platform's portability and speed, establishes it as a transformative step toward integrated, real‐time diagnostic devices for ultrasensitive biomarker detection that can be deployed at the point of need, shortening the timeline from testing to treatment.

## 2 Results

### 2.1 Working Principle

**FIGURE 1:**
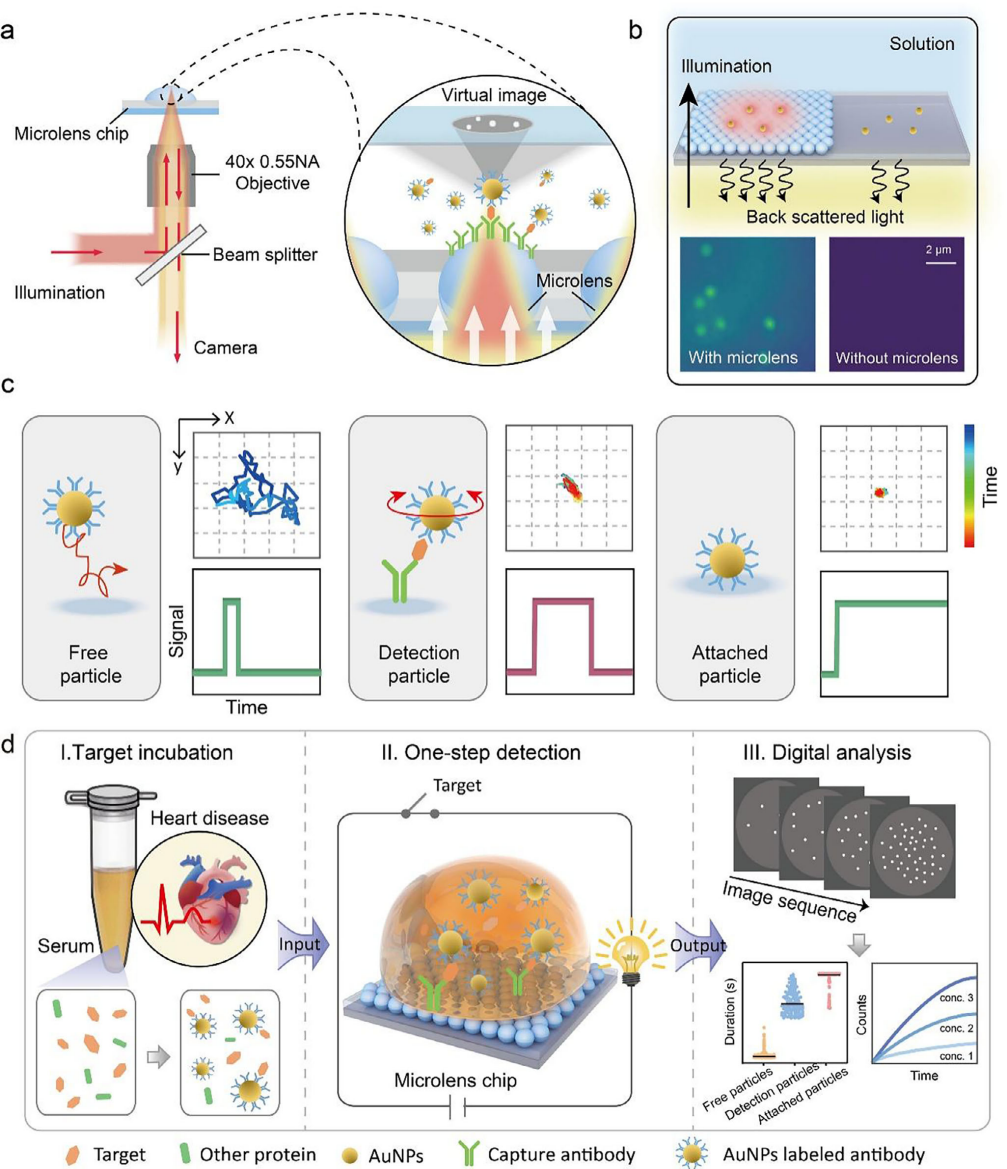
Schematic illustration of the dynamic immunoassay for single‐binding events profiling. (a). The microlens chip acts as a sensing surface and superlens layer for dynamic measurement of molecular interactions under a conventional inverted microscopic setup. The enlarged view on the right side demonstrates the formation of a binding unit on the microlens chip surface through a sandwich reaction, resulting in a magnified virtual image of AuNPs for quantification. (b). Upon illumination, the microlens collects the scattered light emitted by the individual 80 nm AuNPs, generating a bright spot with high contrast. However, the single AuNPs are rarely visible without the assistance of the microlens. (c). The captured AuNPs are classified into three categories: free particles, attached particles, and detection particles according to their trajectories. (d). A heart disease‐related biomarker is quantified via specific recognition in one‐step detection. Nonspecific binding events, including free and attached particles, are excluded from the digital analysis without washing steps. The detection particles are considered as target‐positive specific binding events.

Our single‐binding event profiling approach employs plasmonic AuNPs as molecular labels in a typical sandwich assay format, enabling precise digital counting of biomolecular interactions (Figure 1a). When target molecules are present in the sample, they form “AuNP‐labeled antibody – target – capture antibody” immunoconjugates on the microlens chip surface. The dynamic binding of AuNPs to the surface is directly correlated with target presence, facilitating high‐sensitivity detection of individual binding events. Enabling real‐time detection of this dynamic binding process, we integrate a unique microlens chip into a portable optical imaging system. This chip, composed of a barium titanate glass dielectric microsphere array, enhances the backscattering of visible light from individual AuNP labels. This enhancement arises from amplified long‐range optical fields and complex composite interactions between the AuNPs and the closely spaced microspheres, spanning near‐field to semi‐near‐field regimes. Crucially, this generates a sufficient number of detectable photons and a high signal‐to‐noise ratio (SNR), enabling imaging resolution beyond the diffraction limit and high‐performance scattering‐based single‐particle tracking (S‐SPT) even under a conventional bright‐field microscopic optical path with an incoherent white LED source [28, 29, 30]. This S‐SPT capability effectively circumvents the diffraction limit inherent to our low‐NA objective lens (40×, NA = 0.55). As a result, individual 80 nm AuNPs, which are otherwise invisible without the microlens (Figure 1b), generate clearly resolvable, high‐contrast bright spots. This microlens‐mediated enhancement facilitates fast and efficient light‐scattering imaging without requiring complex optics or tedious image processing. Once captured, individual AuNPs are analyzed in real time for positional localization and motion tracking. Based on their trajectories, the AuNPs are classified into three distinct categories (Figure 1c): (i) free particles that move freely in solution via Brownian motion and do not remain visible across consecutive frames; (ii) detection particles that exhibit restricted Brownian motion, indicating successful target recognition via the sandwich immunoassay; and (iii) attached particles that remain motionless due to nonspecific adsorption onto the chip surface, contributing to background noise. By leveraging these inherent kinetic characteristics, we can automatically exclude nonspecific binding events (free and attached particles) without requiring washing steps. This ensures that only true detection events are counted in real time.

For quantitative digital analysis, the effective microlens‐assisted imaging region of each microsphere was defined as the region within 70% of the physical radius of the microsphere and used as the unit area for AuNP enumeration. The net count of detection particles per unit area provides a highly specific and ultrasensitive biomarker quantification method. This approach holds great potential for clinical applications, particularly in cardiovascular disease diagnostics, where rapid and precise detection of biomarkers such as cTnI is critical (Figure 1d).

### 2.2 Enhanced Detection of Individual Plasmonic Nanoparticles by On‐Chip Microlens

Although AuNPs are renowned for their excellent optical properties and efficiency as plasmonic labels for single‐signal readout, visualizing and detecting them at the single‐particle level has traditionally required advanced optical systems, such as dark‐field imaging, interferometric detection, and SPR microscopy. Moreover, these techniques have relied exclusively on high‐NA lenses, which inherently limit the FOV [31].

**FIGURE 2:**
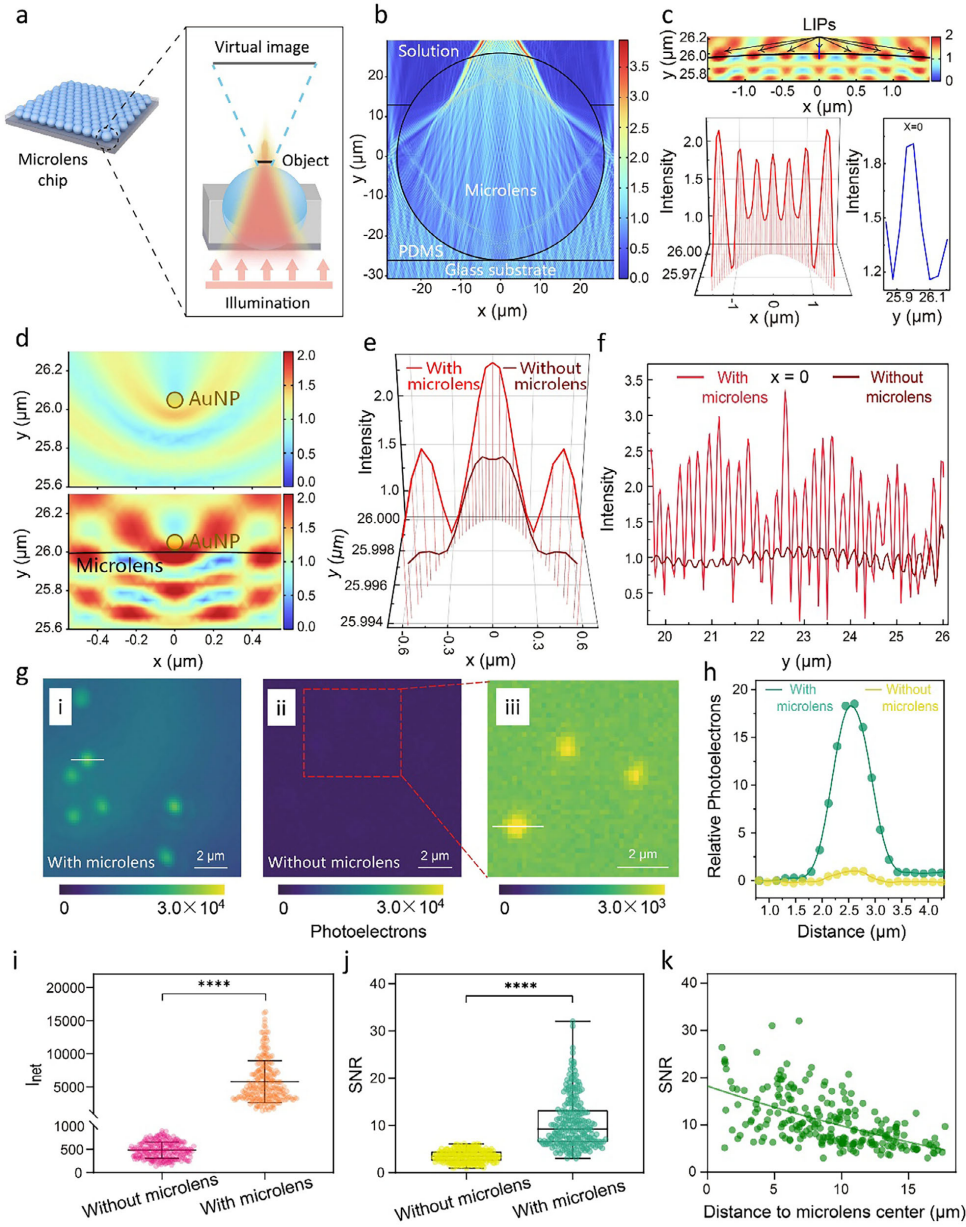
The on‐chip enhancement of individual plasmonic nanoparticles by the microlens chip. (a). Schematic illustration of the enhancement of the microlens chip. Each microlens within the microlens chip functions as an auxiliary lens, projecting a magnified virtual image of the object into the far field. (b). Simulation of the electric field (|E|) distribution generated by the microlens using the finite element method. The diameter of the microlens is 52 µm, and the wavelength of the illumination light is 630 nm. (c). Simulation of the localized intensity peaks (LIPs) in the near field of microspheres in the x‐direction and y‐direction. (d). LIPs simulation showing the intensity distribution around AuNPs without (top panel) or with (bottom panel) the presence of the microlens. The AuNPs with a diameter of 80 nm are positioned on the chip surface. (e). Peak intensity extracted from (d) in the x‐direction and y‐direction. (f). Peak intensity of the simulated result of (d) in the y‐direction while *x* = 0. (g). Experimental photoelectron images of 80 nm diameter AuNPs imaged with (i) and without (ii and iii) the use of a microlens chip. The photoelectron data are acquired with an exposure time of 1 ms with white LED light illumination (power intensity = 0.7 W cm^−2^). Note that for the convenience of visual identification, the scale bars in i and iii are different. (h). Comparison of their relative number of photoelectrons from individual AuNPs in (g). Their photoelectrons are normalized by the maximum value of the AuNP imaged without the microlens chip. (i). Quantitative comparison of the net intensity (I_net_) of individual AuNPs imaged with and without the microlens chip. Horizontal lines and error bars denote mean ± SD. (j). Quantitative comparison of the signal‐to‐noise ratio (SNR) of individual AuNPs imaged with and without the microlens chip. The center line and box denote the median and interquartile range, respectively, and whiskers indicate the data spread. (k). Spatial dependence of the SNR of individual AuNPs within the effective microlens‐assisted imaging region. The solid curve represents a nonlinear fit, showing that the SNR decreases with increasing distance from the microlens center. For panels (i–k), each dot represents one AuNP. A total of 230 AuNPs were analyzed under identical optical conditions. In the microlens‐assisted group, the analyzed AuNPs were collected from the effective microlens‐assisted imaging regions of 26 individual microlenses. ^****^, *p* < 0.0001.

In contrast, we have developed a direct and efficient on‐chip signal enhancement strategy that facilitates the observation of individual plasmonic nanoparticles using a simpler optical system with a low‐NA objective lens. Central to our approach is a microlens chip composed of a densely packed array of high‐refractive‐index dielectric microspheres. These microspheres amplify the scattering intensity from individual AuNPs (Figure 2a). The dielectric microspheres produce a magnified virtual image of the AuNPs in the far field, which is then captured by the objective lens. In addition, the self‐assembly of the microspheres into a microarray on a substrate ensures that this enhancement platform is both easy to fabricate and robust.

To illustrate the light‐scattering enhancement provided by the microspheres, we performed finite element method simulations of the electric field distribution for a microlens with a 52 µm diameter (Figure 2b). The simulations reveal that the illumination light is strongly focused and enhanced near the rear surface of the microsphere. Figure 2c further examines the localized intensity peaks (LIPs) in two‐dimensional near‐field domains, showing that these peaks arise from the microsphere‐induced focusing effect and lead to a pronounced amplification of the local electric field. Under identical simulation parameters, we compared the intensity distribution around plasmonic AuNPs with and without the microlens (Figure 2d). The results clearly show a significant enhancement in the backscattered light intensity when the microsphere is present (Figure 2e), while in its absence the intensity in the y‐direction remains nearly constant, indicating little or no enhancement (Figure 2f). Notably, the degree of field enhancement in our system is primarily determined by the refractive index and size of the microspheres. These results suggest that the microlens enhances the detectability of individual AuNPs through the coupled effects of efficient focusing of the illumination light and localized electric‐field amplification, which together increase the backscattered signal and improve image contrast.

We experimentally validated these findings by capturing bright‐field microscopy images of 80 nm AuNPs with and without the microlens chip under identical illumination conditions. Consistent with our simulations, AuNPs imaged with the microlens exhibited pronounced backscattering enhancement – appearing as distinct, bright, spot‐like point spread functions against the background – while images acquired without the microlens showed only subtle intensity variations (Figure 2g). To quantitatively assess the scattering enhancement effect, we defined an enhancement factor δ as δ = N_PE‐µlens_/N_PE_, where N_PE‐µlens_ and N_PE_ represent the integrated net photoelectrons detected with and without the microlens, respectively. Our measurements indicate that the microlens chip increases the number of photoelectrons from individual AuNPs by approximately 13‐fold under identical illumination conditions (Figure 2h). This substantial improvement in light‐scattering intensity allows for the straightforward observation of plasmonic AuNPs at the single‐particle level in bright‐field imaging, eliminating the need for complex image processing algorithms.

To further quantify the imaging performance under identical optical conditions, we statistically analyzed 230 individual AuNPs imaged with and without the microlens chip. Consistent with the photoelectron‐based enhancement analysis, the mean net intensity of individual AuNPs increased from 480.5 to 5786 in the presence of the microlens chip, corresponding to an approximately 12.0‐fold enhancement (Figure 2i). In parallel, the mean SNR rose from 3.48 to 10.62, representing an approximately 3.05‐fold improvement (Figure 2j), indicating that the microlens chip enhances not only the signal level but also the detectability of individual AuNPs over the background. Detailed definitions of net intensity and SNR are provided in the Supporting Information. To assess the FOV statistics of this enhancement, we further examined the spatial dependence of SNR within the effective microlens‐assisted imaging region. As shown in Figure 2k, the SNR of individual AuNPs gradually decreased with increasing distance from the microlens center, confirming that the strongest enhancement occurs near the central region of the effective microlens‐assisted imaging area. Nevertheless, within the effective imaging region, individual AuNPs remained clearly distinguishable from the background and could be readily resolved. This spatial trend is consistent with the simulated optical field distribution and localized intensity enhancement induced by the microsphere. Additionally, these statistics were collected from the effective microlens‐assisted imaging regions of 26 individual microlenses, demonstrating that the enhancement effect is robust and reproducible across multiple microlenses.

Importantly, this on‐chip signal enhancement greatly reduces the demands on the optical system. Rather than relying on high‐magnification and high‐NA objectives, our approach employs a conventional inverted optical microscopic setup equipped with a 40× low‐NA (0.55) objective lens and an unpolarized, incoherent white LED light source. Under these conditions, single AuNPs as small as 60 nm can be clearly visualized (Figure S5). To our knowledge, this is the first report of such small AuNPs being observed with high temporal resolution using bright‐field white‐light microscopy. For our experiments, we selected microspheres with a diameter of 52 µm and AuNPs of 80 nm to achieve a satisfactory SNR while minimizing particle sedimentation during the immunoassay (Figure S6).

It is well established that using a low‐magnification objective lens expands the FOV, thereby increasing the region of interest (ROI). For instance, the detection area increases to approximately 0.11, 0.44, and 1.77 mm^2^ when using 40×, 20×, and 10× objectives, respectively. To assess the feasibility of lower‐magnification imaging, 80 nm AuNPs on the chip surface were comparatively imaged using 10×, 20×, and 40× objectives (Figure S7). We observed that individual AuNPs visible under the 40× objective remained discernible with a 20× objective lens (NA = 0.4), provided that sufficient spatial separation existed between particles. Although low‐magnification objectives may occasionally introduce counting inaccuracies due to closely spaced particle pairs, under ultralow biomarker concentration, where particles are sparsely distributed, this error is minimal. Furthermore, a larger detection area contributes to an improved limit of detection (LOD), as the LOD is inversely proportional to the detection area [11]. Consequently, our approach shows promising potential for detecting biomarkers at ultralow concentrations without the need for complex mechanical or optical scanning to enlarge the ROI.

This on‐chip microlens strategy not only enhances the detection of individual plasmonic nanoparticles but also simplifies the optical system required for high‐temporal‐resolution measurements. This provides an alternative to conventional time‐resolved biosensing techniques that depend on high‐NA optics, and it paves the way for practical, low‐cost, and miniaturized diagnostic devices capable of ultrahigh‐sensitivity detection of rare biomarkers.

### 2.3 Recognition and Profiling of Single‐Binding Events

Utilizing microlens‐enhanced light‐scattering imaging, the dynamic binding of individual AuNPs on the chip surface becomes clearly visible, and their positions are accurately recorded by a complementary‐metal‐oxide‐semiconductor (CMOS) sensor. To demonstrate dynamic detection directly correlated with the binding of AuNPs, we employed cTnI as a proof‐of‐concept target. The sensing region of the microlens chip was precoated with capture antibodies via covalent binding. The sample containing cTnI was incubated with AuNP‐labeled antibodies for 10 min. This pre‐formed mixture was then applied to the chip, where target‐specific interactions generated AuNP‐labeled binding events. Utilizing microlens‐enhanced imaging, our portable device recorded bright‐field video at 500 frames per second, enabling the dynamic binding of individual AuNPs to be clearly visualized and their positions accurately tracked by the CMOS sensor. This high‐speed imaging was primarily used to reveal the intrinsic kinetic features of each particle type, thereby establishing the physical basis for distinguishing different particle states at the single‐event level.

**FIGURE 3:**
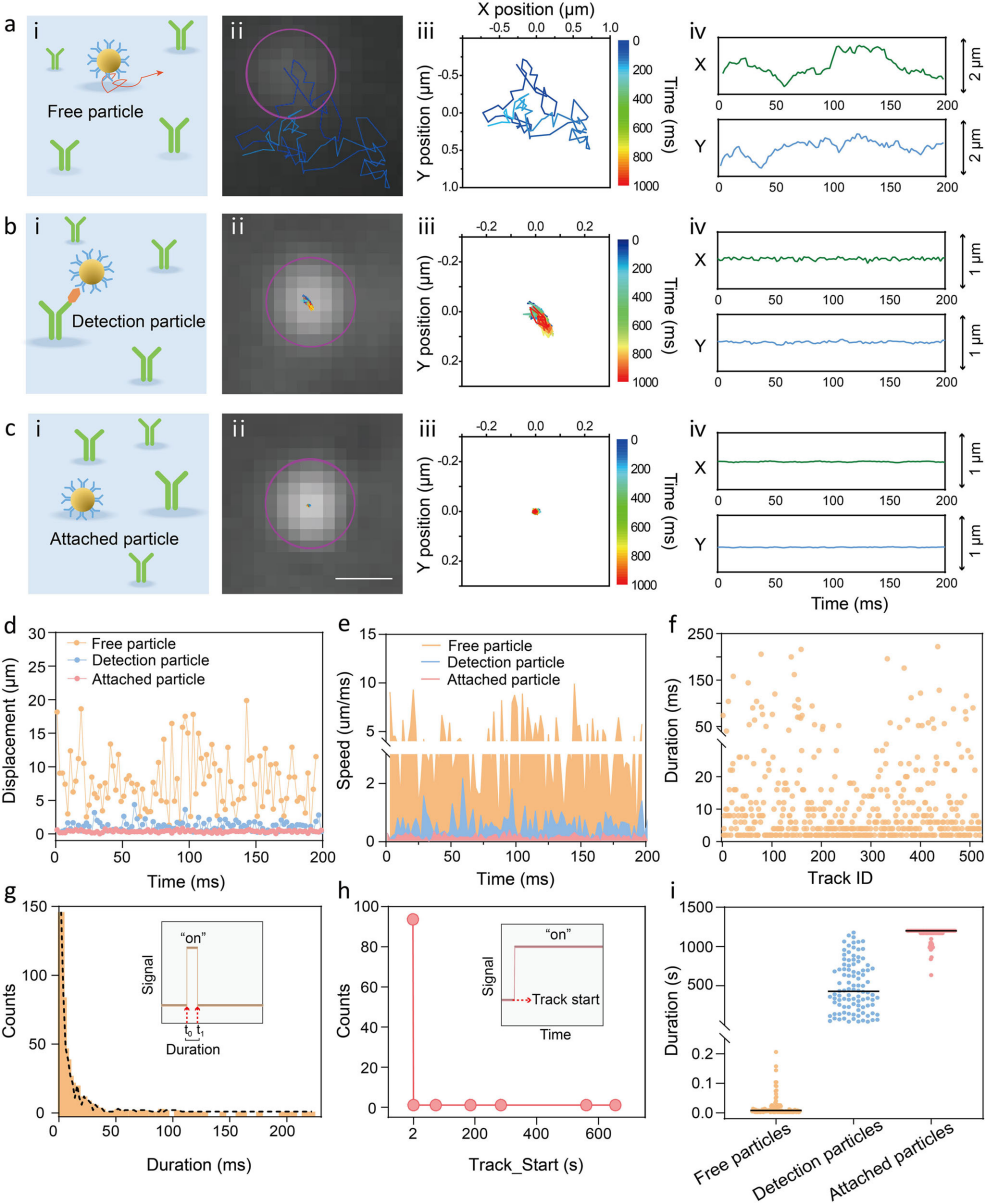
Single‐particle recognition. According to the binding and unbinding events, the captured AuNPs are classified into three patterns: Free particles (a), Detection particles (b), and Attached particles (c). The shown schematic illustrations (i), typical bright‐field images acquired at 500 fps for 1 s (ii), the representative motion patterns with color indicating time from 0 to 1 s (iii), and the time traces in the x and y directions (iv) are derived from the same representative particles on the chip surface. The purple circles are used to show the location of the nanoparticles, and the scale bar is 2 µm in (ii). (d). Displacement of three representative particles between two consecutive frame points over time. (e). Speed of three representative particles over time. Note that the results in (d, e) are recorded from the same particles in (a–c) within 200 ms at 500 fps. (f). Duration of free particles from tracking 520 particles at 500 fps for 1 s. The track ID corresponds to these free particles. (g). Distribution of duration of free particles in (f). The insert shows the duration refers to the time period from signal “on” (t_0_) to “off” (t_1_). (h). Distribution of start track of attached particles from counting 100 particles in the absence of target. The insert indicates the start of track refers to the time beginning from signal “on”. (i). Duration of three types of particles by counting 100 particles of each type, respectively. Specifically, the attached and detection particles were tracked for 19 min with an interval of 2 s to further characterize their duration. The black line represents the average value of the duration of 100 particles.

The captured AuNPs displayed three representative motion patterns that were analyzed through direct positional localization and motion tracking (Figure 3a–c). Based on their trajectories, the particles were classified into three distinct categories: free particles (Figures 3a‐i), detection particles (Figure 3b‐i), and attached particles (Figure 3c‐i). Free particles, which diffuse in solution, exhibit Brownian motion with random fluctuation within approximately 2 µm in both the *x* and *y* directions (Figure 3a‐iii,iv). In contrast, detection particles, i.e., target‐positive, show restricted Brownian motion characterized by a more polarized trajectory (Figure 3b‐iii), consistent with previous studies [32, 33]. Their time traces revealed slight distance fluctuations along the x and y directions attributed to a tethering effect between the particle and the surface (Figure 3b‐iv). Attached particles, which remain almost motionless because of nonspecific adsorption (primarily due to electrostatic interactions), contribute to background noise (Figure 3c‐iii,iv).

To further characterize the dynamic behavior of these particles, we recorded their displacement and speed over time (Figure 3d,e). A sequential reduction in variability among free, detection, and attached particles over a 200 ms interval was accompanied by a corresponding decrease in displacement and speed. This trend enabled clear recognition of the distinct states of single‐binding events based on particle motion within the effective microlens‐assisted imaging region.

More importantly, the three particle types exhibit unique kinetic features associated with their motion patterns. In our system, particles are only captured when they diffuse into or are drawn to the microlens chip surface within its focal depth. Here, the “duration” is defined as the bound lifetime, i.e., from the moment the signal turns “on” (binding) until it turns “off” (unbinding). Free particles, governed by Brownian motion, were transiently captured as they randomly diffused near the chip surface and then quickly disappeared. As shown in Figure 3f, the duration of free particles was predominantly within 250 ms (based on tracking 520 particles for 1 s), with approximately 92% of them exhibiting durations of less than 50 ms (Figure 3g). In contrast, attached particles, driven mainly by strong electrostatic forces, were rapidly immobilized on the chip surface, constituting a major source of background noise. Analysis of the initial binding (“track start”) for attached particles revealed that about 96% of them attached within the first 2 s, based on tracking 100 particles in the absence of target (Figure 3h). While the 500 fps short‐term imaging established the kinetic signatures of the three particle states, long‐term bright‐field image sequences acquired over 19 min at 2 s intervals were used to evaluate frame persistence and appearance time for practical digital counting, with the first frame defined as 0 s. Detection particles, i.e., those specifically bound to the target, remained visible throughout the entire immunoassay. Their duration distributions spanned different time periods (Figure 3i), enabling precise recognition and profiling of single‐binding events.

This dynamic profiling approach provides a kinetic basis for distinguishing transient from stable interactions, enabling high‐temporal‐resolution analysis of molecular interactions while reducing background interference. By integrating on‐chip microlens enhancement with backscattering‐enhanced S‐SPT, our method resolves the motion features and residence behavior of individual AuNPs under conventional bright‐field microscopy. This dual capability delivers the exceptional spatial resolution required to track constrained Brownian motion and the high temporal resolution necessary for kinetic characterization of binding events.

### 2.4 Dynamic Tracking of Binding Events Over Time

**FIGURE 4:**
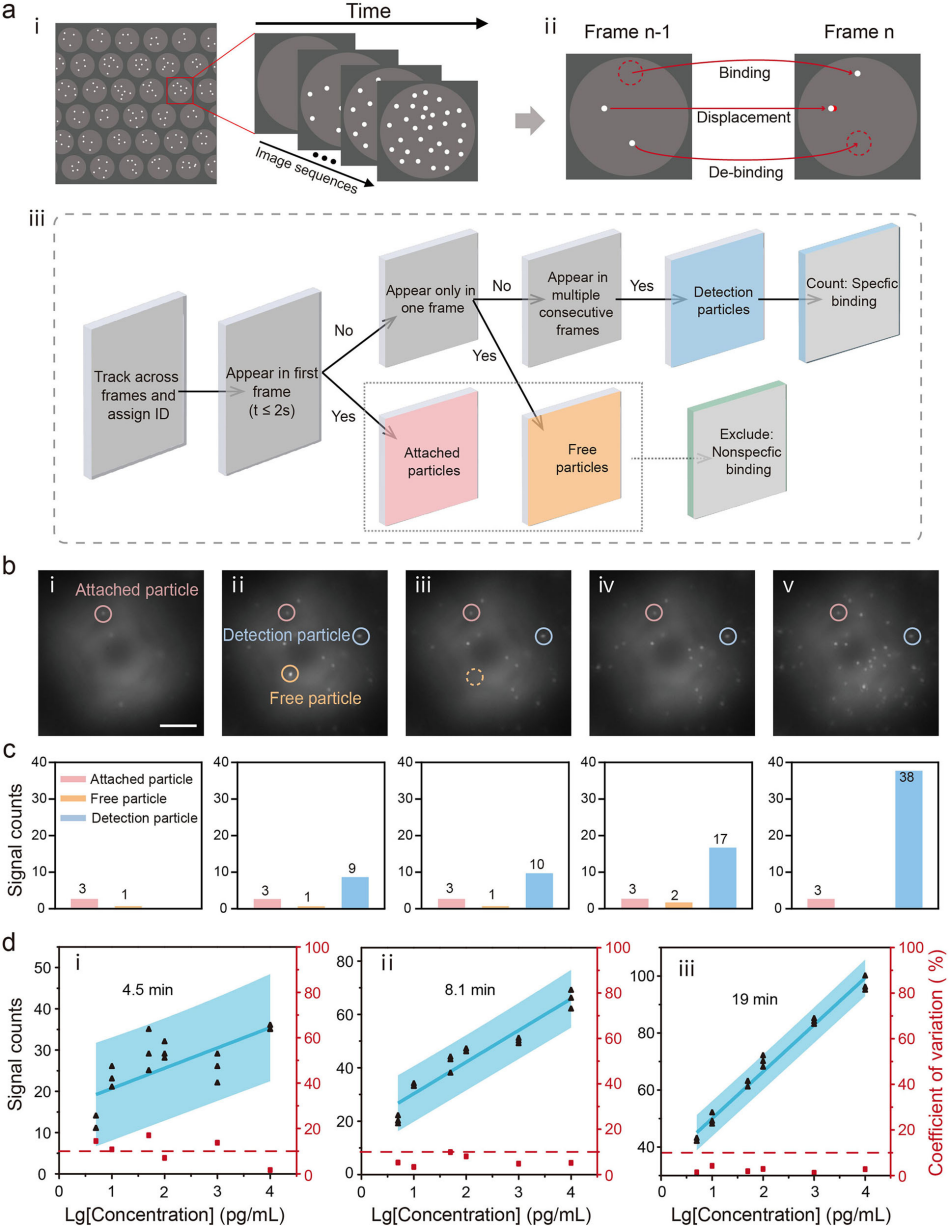
Real‐time tracking and classification of single‐binding events. (a). Diagram of the multistep dynamic tracking workflow. (i): Select a region of interest in the acquired bright‐field images and detect particles in the image sequences. (ii): Compare particle positions between sequential frames for particle localization. (iii): Decision logic for particle classification and digital counting. For time‐resolved specific counting, consecutive frames were acquired at 2 s intervals. Particles with assigned track IDs were classified as free, attached, or detection particles according to their frame persistence and appearance time. Nonspecific binding events (free and attached particles) were excluded, whereas only specific binding events (detection particles) were counted for quantification. (b). Typical bright‐field images recording nonspecific and specific binding events in one statistical unit at different time points: 0 s (i), 362 s (ii), 364 s (iii), 600 s (iv), and 1136 s (v). Scale bar = 10 µm. (c). Digital AuNP counts calculated from the corresponding bright‐field images in (b). (d). Digital signal counts (black) and coefficient of variation (CV, red) for various cardiac troponin I (cTnI) concentrations at counting intervals for 4.5 min (i), 8.1 min (ii), and 19 min (iii). Black triangles indicate digital signal counts independently obtained from three replicate microsphere‐based statistical units. Solid blue line: linear fit to the data. Light‐blue region: 95% prediction interval based on the measurements. Red squares: corresponding CV. Dashed red line: CV = 10%.

Since single‐molecule measurements rely on the accumulation of discrete binding events, accurately enumerating these events is critical for reliable quantification. However, distinguishing specific binding events from a background of nonspecific interactions is technically challenging. In this study, dynamic profiling refers to the time‐resolved on‐chip tracking, kinetic‐based classification, and digital counting of individual AuNP‐associated binding events during the post‐loading observation window. Each binding event is directly associated with the binding of AuNPs. At the same time, unbound or nonspecifically bound AuNPs, i.e., free and attached particles, are also captured within the effective microlens‐assisted imaging region, potentially contributing to false‐positive signals. By precisely recognizing and profiling individual binding events, this dynamic tracking method effectively distinguishes and excludes these false positives through digital analysis (Figure 4a), enabling accurate real‐time enumeration of specific binding events.

Briefly, bright‐field image sequences were continuously recorded under a 40× low‐NA objective, and particles within a selected ROI were first detected from the image sequence (Figure 4a‐i). Next, particle positions were compared between consecutive frames for precise localization (Figure 4a‐ii): particles appearing in sequential frames with minimal displacement were linked and assigned a consistent track ID. Building on the kinetic features revealed by high‐speed imaging, we defined a decision logic for long‐term digital analysis at 2 s intervals that applies time‐resolved classification rules (Figure 4a‐iii). Particles present in the first frame or appearing within the first 2 s were classified as attached, matching our observation that approximately 96% of attached particles appeared within this window in the absence of target. Particles observed in only a single frame were classified as free, because high‐speed tracking established that free particles persist for less than 250 ms, a duration much shorter than the 2 s acquisition interval. Consequently, nonspecific events, comprising free and attached particles, were efficiently excluded without washing steps typically required in conventional methods. The particles that survived beyond this initial exclusion window were classified as detection particles, representing specific target‐associated binding events, and were digitally counted for target quantification. Figure 4b shows a series of representative images where nonspecific and specific binding events are tracked over time. The corresponding digital AuNP counts in Figure 4c further demonstrate that free, attached, and detection particles can be precisely enumerated in each frame (Figure 4c). This digital analysis allowed us to plot the cumulative number of specific binding events over time, thereby improving the accuracy in distinguishing specific from nonspecific binding and simplifying the assay protocol to a single step.

To further evaluate how potential misclassification may influence counting accuracy, we performed a quantitative robustness analysis of the kinetic‐based decision logic, with detailed results provided in Figure S8. Free particles appeared only transiently within the microlens focal depth (< 250 ms), typically within a single frame, and were therefore efficiently excluded during trajectory tracking under the 2 s acquisition interval; their contribution to false‐positive detection counts was negligible. The major potential source of misclassification was instead associated with initially attached particles. We therefore varied the attached‐particle exclusion window from 0 to 10 s. The results demonstrate that a 2 s exclusion window markedly reduced the blank count while preserving the low‐concentration positive signal, whereas longer windows provided only marginal additional background reduction but gradually decreased the retained positive counts. This threshold‐dependent behavior indicates that the selected 2 s window provides a practical balance between false‐positive suppression and true‐signal retention, with limited influence of potential misclassification on counting accuracy and analytical performance. A detailed quantitative analysis is provided in the Supporting Information. Moreover, dynamic tracking of binding events in real time aids in optimizing detection time, achieving acceptable accuracy and detection limits without waiting for the immunoassay to reach equilibrium. This approach also significantly reduces counting errors caused by the accumulation of excessive AuNPs in regions smaller than the optical resolution limit. As illustrated in Figure 4d, we investigated detection accuracy, represented by the coefficient of variation (CV), plotted against the measured signal counts for cTnI concentrations ranging from 5 to 10 000 pg mL^−1^, with counting times of 4.5, 8.1, and 19 min, respectively. Initially, within 4.5 min, the limited number of counted AuNPs resulted in scattered data and a higher CV. As detection time increased, the higher number of AuNP counts led to more consistent data and a gradual reduction in the CV. Notably, the CV dropped below 10% after 8.1 min, indicating that this is the minimal reliable detection time. Prolonging the detection time beyond 19 min did not further improve the CV, so 19 min was selected as the optimal on‐chip counting window for PBS‐based cTnI quantification.

This dynamic tracking approach, combined with microlens‐enhanced imaging, offers a practical strategy for wash‐free digital biosensing. By leveraging kinetic features and time‐resolved classification rules to discriminate specific binding events from background noise in real time without the need for extensive washing steps or high‐NA optics, our method simplifies the assay workflow while enhancing both accuracy and sensitivity for ultralow biomarker detection.

### 2.5 Digital Immunoassay for cTnI Quantification

**FIGURE 5:**
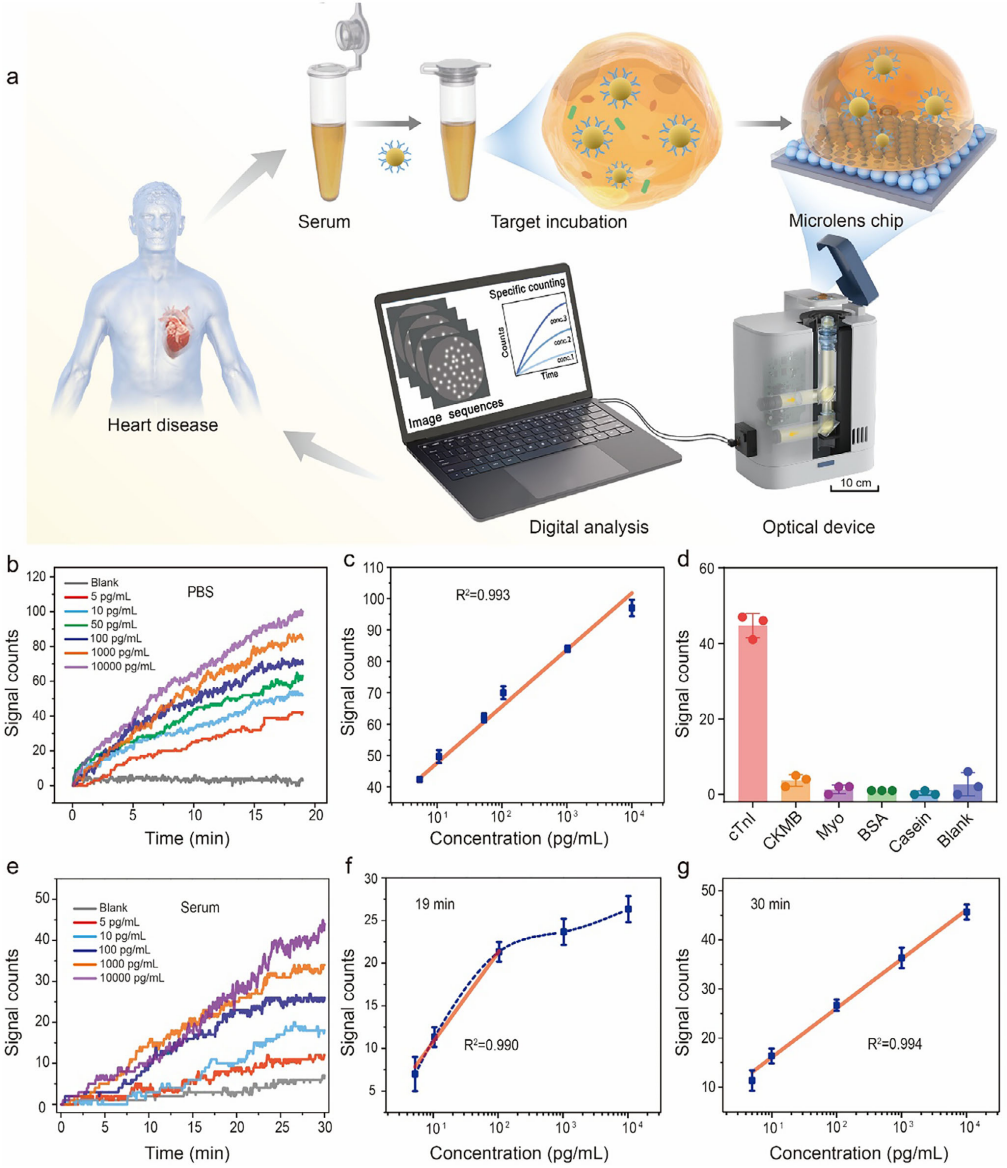
One‐step cTnI detection using a portable optical device. (a). Schematic illustration. (b). Representative time‐course traces of digital signal counts from one microsphere‐based statistical unit for cTnI in PBS at 0 (blank), 5, 10, 50, 100, 1000, and 10 000 pg mL^−1^. (c). Signal counts exhibit a linear relationship with the logarithms of cTnI concentrations in PBS within the range of 5–10 000 pg mL^−1^ at 19 min. (d). Specificity of the digital immunoassay against cTnI (5 pg mL^−1^) and other nonspecific proteins (10 ng mL^−1^). The nonspecific proteins include creatine kinase‐MB (CKMB), myoglobin (Myo), bovine serum albumin (BSA), and casein. (e). Representative time‐course traces of digital signal counts from one microsphere‐based statistical unit for cTnI spiked in serum at 0 (blank), 5, 10, 100, 1000, and 10 000 pg mL^−1^. (f). Signal counts exhibit a linear relationship with the logarithms of cTnI concentrations spiked in serum within the range of 5–100 pg mL^−1^ at 19 min. (g). Signal counts exhibit a linear relationship with the logarithms of cTnI concentrations spiked in serum within the range of 5–10 000 pg mL^−1^ at 30 min. Error bars represent the standard deviation of the counts from three independently analyzed microsphere‐based statistical units.

Leveraging the dynamic tracking capability described above, we first validated the sensitivity and dynamic range for counting single‐binding events using cTnI as a proof‐of‐concept target. Specifically, cTnI detection was performed via a sandwich immunoassay, in which “AuNP‐labeled antibody—cTnI—capture antibody” complexes were formed on the microlens chip surface upon introduction of a sample containing cTnI. The dynamic binding of AuNPs was tracked in real time using a self‐developed portable optical device combined with a microlens chip (Figure 5a). This device is primarily composed of a built‐in optical module with a reflective optical path, an LED light source, a CMOS camera, a sample stage, and a control system (Figure S9). A series of control experiments confirmed that AuNPs bind to the surface only when the complete sandwich immunoconjugates are formed (Figure S10). To assess feasibility, cTnI was spiked into PBS while maintaining a constant concentration of AuNP labels at 10 fM. Figure 5b displays the signal counts of specific binding over a 19‐min period (with a 2‐s acquisition interval) as cTnI concentration increased from 0 (blank) to 10 000 pg mL^−1^. This time‐course trace corresponds to a representative microsphere‐based statistical unit; the replicate‐based total count statistics are provided in Figure S11a. The results clearly show that a higher cTnI level leads to a faster increase in AuNP counts, thereby validating the principle that greater cTnI concentration results in more AuNPs binding to the chip surface. Within the concentration range of 5–10 000 pg mL^−1^, a strong linear correlation was observed between the signal counts and the logarithm of cTnI concentration at 19 min, yielding a high coefficient of determination (R^2^ = 0.993), as demonstrated in Figure 5c. Based on this validated linear range, the calibration curve was fitted as y = 30.38 + 17.90 lg(x), where y is the signal count and x is the cTnI concentration (pg mL^−1^). Using this fitted curve, the LOD and limit of quantitation (LOQ) were estimated as the concentrations corresponding to the blank signal plus three and ten standard deviations of the blank, respectively, yielding values of 0.051 and 0.240 pg mL^−1^. These LOD and LOQ values were obtained by extrapolation below the validated linear range; the experimentally validated linear quantitative range of the assay remains 5–10 000 pg mL^−1^. The estimated LOD is significantly lower than the clinical cutoff levels for healthy individuals (∼ 40 pg mL^−1^) [34, 35], highlighting the method's potential for early diagnosis of AMI. Importantly, compared to other commercial cTnI detection technologies (Table S1), our single‐binding event counting method exhibits superior sensitivity, lower instrument cost, and a simplified one‐step operation. Moreover, by employing microlens‐assisted scattered light imaging, our system uses a simple optical path and a low‐NA objective, which contrasts favorably with other digital immunoassays that require high‐magnification optics (Table S2). This simplification not only reduces the cost of the optical system but also facilitates the development of integrated and miniaturized instruments.

To further evaluate specificity and clinical utility, we applied the method to differentiate interfering proteins and measured cTnI in real serum samples. Interference tests were conducted using creatine kinase‐MB (CKMB), myoglobin (Myo), bovine serum albumin (BSA), and casein. As shown in Figure 5d, the signal counts for the target cTnI group were significantly higher than those for each interfering protein group, even when the concentration of interfering proteins was substantially higher than that of cTnI. In fact, the interference groups exhibited signal counts comparable to the blank, confirming that the method possesses acceptable specificity for cTnI detection. In parallel, we measured various levels of cTnI spiked into undiluted human serum. Similar to the measurements in PBS buffer, signal counts in serum were recorded over 30 min with 2‐s intervals. As the cTnI concentration increased from 5 to 10 000 pg mL^−1^, a gradual increase in signal counts was observed (Figure 5e). To ensure statistical reliability, note that Figure 5e presents a representative time‐course from one microsphere‐based statistical unit, whereas the replicate‐based total count statistics are given in Figure S11b. Notably, within the first 19 min, signal counts maintained a linear relationship with cTnI concentrations in the 5–100 pg mL^−1^ range (Figure 5f). When extending the tracking period to 30 min, the linearity was preserved across the full concentration range of 5–10 000 pg mL^−1^ (Figure 5g). The observed differences between serum and PBS detections are primarily attributed to variations in mass transport dynamics in the solid chip‐based system across different media. Additionally, we calculated a count‐based SNR (SNR_count_) to evaluate low‐concentration counting reliability (Figure S12). Attached‐particle exclusion improved the SNR_count_ in PBS by suppressing false‐positive background events, whereas the SNR_count_ changed only slightly in serum, suggesting that early attached particles were not the major source of serum counting uncertainty. Nevertheless, the signal counts of 5 pg mL^−1^ remained distinguishable from the blank in serum, supporting reliable low‐concentration digital counting under the current acquisition conditions.

The reproducibility of our kinetic decision logic was further validated by comparing the CV of digital counts before and after attached‐particle exclusion (Figure S13). In PBS, the CV remained consistently low across the tested concentration range. In serum, the CV was higher near the detection limit – attributable to reduced event throughput and Poisson fluctuations – but decreased at higher concentrations as binding events accumulated. Moreover, comparison of calibration curves confirmed that the classification logic preserves quantitative fidelity across different matrices with minimal misclassification‐induced bias (Figure S14). Collectively, these results demonstrate that the proposed method enables sensitive detection of low‐concentration targets in real serum samples, while longer detection times are beneficial for higher concentrations to allow sufficient accumulation of binding events for accurate quantification.

Overall, this digital immunoassay based on single‐binding event counting, enhanced by an on‐chip microlens, provides a highly sensitive, cost‐effective, and straightforward platform for cTnI quantification. By reducing reliance on high‐NA optics and simplifying the detection protocol into a single‐step on‐chip operation, our approach supports time‐resolved biosensing in a compact optical format, and highlights the potential of the platform for practical, miniaturized biosensing applications.

## 3 Discussion

Single‐molecule kinetic assays represent a transformative strategy to overcome the limitations of endpoint assays, particularly those imposed by limited probe affinity and nonspecific binding. However, the broader adoption of kinetic assays has been hampered by their reliance on sophisticated instruments and highly efficient probes to enable real‐time monitoring with nanoscale precision. To address these challenges, we developed a simple yet powerful platform for real‐time visualization and enumeration of single‐binding events by enhancing the detection of plasmonic AuNPs via an on‐chip microlens array.

In our system, the microlens chip acts as a superlens layer that efficiently amplifies the light scattering intensity of individual AuNP labels, facilitating direct localization and dynamic tracking of individual AuNPs under a simple bright‐field optical system with low‐NA objectives. This strategy effectively offers the spatial resolution required to precisely visualize confined Brownian motion and the temporal resolution necessary to track dynamic binding events in real time. Moreover, by harnessing microlens‐enhanced imaging, the method substantially reduces the dependency on advanced and sophisticated optical setups for single‐nanoparticle readout, paving the way for developing integrated and miniaturized optical devices that are suitable for real‐time diagnostics.

By leveraging the distinct kinetic fingerprints of individual binding events, our method enables precise discrimination between specific and nonspecific interactions, this is a capability central to high‐fidelity biosensing. Importantly, nonspecifically bound AuNPs, which contribute a false‐positive signal in an endpoint readout, can be effectively identified and eliminated through digital analysis without resorting to time‐consuming washing steps, thereby allowing for reliable tracking and enumeration of true binding events over time in complex matrices. As demonstrated by cTnI detection, the proposed method exhibits an extensive linear range (5–10 000 pg mL^−1^), a low detection limit (0.051 pg mL^−1^), great selectivity, and practical applicability in clinical samples. Looking forward, ongoing advancements in microfluidics offer a promising opportunity to further enhance our system. Integrating microfluidic technology into the current method will improve mass transport in solid chip‐based systems when working with clinical samples, thereby reducing the assay time for detecting low‐abundance targets [12]. Such integration also improves the scalability and applicability of this system, supporting multiplexed assay designs and the further development of compact optical devices with potential for point‐of‐care translation. Additionally, because the sensitivity of single‐binding event detection is intrinsically associated with the FOV, future improvements may involve the use of lower‐magnification objectives, such as 20× or even 10×, to expand the FOV and capture a larger number of binding events in a single measurement. Enhanced digital analysis across such a wide FOV could further boost both the sensitivity and accuracy of detection [36], laying a solid foundation for the identification of ultra‐low‐concentration biomarkers. Furthermore, beyond protein biomarker detection, this platform holds promise for real‐time analysis of a broad range of molecular interactions, offering insights into binding kinetics and affinity landscapes with unprecedented resolution. For instance, it could be leveraged to map the affinity landscape of molecular libraries by rapidly screening and categorizing probes based on their distinct kinetic fingerprints, thus offering richer mechanistic insights than conventional endpoint assays.

In conclusion, this work demonstrates a microlens‐enhanced biosensing platform that brings single‐binding‐event analysis and kinetic discrimination to a compact and accessible format. This capability underscores its potential to enable robust, real‐time biomolecular interaction studies outside specialized laboratories, facilitating its future development toward point‐of‐care diagnostic applications in precision medicine.

## 4 Materials and Methods

### 4.1 Materials

High‐refractive‐index glass microspheres (52 µm in diameter, refractive index 1.92) were purchased from Microspheres‐Nanospheres (Cold Spring, NY, USA). Citrate‐stabilized AuNPs were purchased from BBI Solutions (Crumlin, UK). Polydimethylsiloxane (PDMS, SYLGARD 184) was obtained from Dow Corning (USA). (3‐aminopropyl) triethoxysilane (APTES), glutaraldehyde (GA, 50%) and SH‐PEG‐NHS (PEG) were purchased from Aladdin Bio‐Chem Technology (Shanghai, China). PBS buffer (1×) was purchased from Sigma–Aldrich Co., Ltd. (St. Louis, MO, USA). Recombinant human cTnI, anti‐cTnI detection antibody, and capture antibody were produced by OriGene Biotechnology Co., Ltd. (Wuxi, China). All reagents were analytical grade and used as received; solutions were prepared with deionized water from a Millipore system.

### 4.2 Preparation of AuNP‐Labeled Antibody

The AuNP‐labeled detection antibody, i.e., the conjugation of anti‐cTnI detection antibody to AuNP, was prepared according to previous work with slight modifications [37]. First, 500 µL of an 80 nm AuNP suspension was stirred gently at room temperature while 20 µg of PEG was added. After 30 min, the thiol group of PEG had covalently anchored the polymer to the gold surface. Next, 10 µg of anti‐cTnI detection antibody was introduced, and the mixture was incubated for 4 h at 37°C with 500 rpm agitation. During this step, the terminal NHS ester of the PEG reacted with the primary amine on the antibody to form a stable amide bond. The resulting conjugates were isolated by centrifugation at 1500 rpm for 20 min, washed three times with deionized water under the same centrifugal conditions, and finally resuspended in ultrapure water for immediate use. Characterization data are provided in Figure S1.

### 4.3 Fabrication of the Microlens Chip

The microlens chip, whose self‐assembled monolayer of dielectric microspheres functions as a superlens, was fabricated as follows. A glass slide was first cleaned with absolute ethanol and deionized water, then activated for 2 min in an oxygen plasma to create a hydrophilic surface. 30 µL of an aqueous microsphere suspension was dropped onto the slide, where capillary forces drove the formation of a close‐packed monolayer. After the surface had dried at 70°C for 10 min, a 10:1 PDMS mixture was spin‐coated at 5000 rpm for 5 min. The thickness was adjusted so that the microspheres remained partially exposed. Trapped air was eliminated under vacuum, and the assembly was cured at 80°C for 1 h. A pre‐cured 500 µm‐thick PDMS film with five punched reaction wells was finally bonded to the array, completing the chip. Figure S2 illustrates the self‐assembly process, while Figure S3 shows the finished structure.

### 4.4 Surface Modification of the Microlens Chip

Chemical functionalization began with a brief oxygen‐plasma treatment to generate surface hydroxyl groups [38]. 100 µL of 5% (v/v) APTES in absolute ethanol was applied for 30 min at room temperature, after which the slide was rinsed three times with absolute ethanol and blown dry with nitrogen. A 1 h bake at 70°C completed silanization. After that, 100 µL of 2.5% (v/v) GA in PBS was introduced for 1 h at room temperature to create aldehyde‐terminated linkers. The anti‐cTnI capture antibody was then immobilized through Schiff‐base coupling between its amine groups and the surface aldehydes. After rinsing with water, nonspecific binding sites were blocked with 2% (w/v) BSA in deionized water. The surface‐modified chip was stored at 4°C and is depicted in Figure S4.

### 4.5 Detection of cTnI

For each measurement, 10 µL of sample containing cTnI was combined with 50 µL of the AuNP‐labeled antibody suspension and gently stirred for 10 min at room temperature before loading onto the microlens chip. The entire mixture was then dispensed into a single reaction well of the microlens chip, enabling one‐step formation of the sandwich “capture antibody–cTnI–AuNP‐labeled antibody” complex. Unless otherwise specified, the on‐chip counting time used for cTnI quantification was 19 min for PBS measurements and 30 min for serum measurements covering the full concentration range. In the serum measurements, the human serum used in this study was derived from human subjects. The use of human‐derived samples was approved by the Institutional Review Board (IRB) of Shenzhen Institutes of Advanced Technology, Chinese Academy of Sciences (SIAT‐IRB‐230715‐H0667).

### 4.6 Image Acquisition and Digital Analysis

Unless otherwise specified, all binding‐event imaging and quantitative analyses in this work were performed using a self‐developed optical device equipped with a 40× objective (NA = 0.55) and LED illumination. A CMOS camera captured images at 500 fps for single‐particle tracking, and at one frame per 2 s for cTnI quantification. For quantitative digital analysis, the effective microlens‐assisted imaging region of each microsphere was defined as the region within 70% of the physical radius of the microsphere and used as the unit area for AuNP enumeration. Each such microsphere‐based unit area was treated as one statistical unit. For the concentration‐level analysis shown in Figures 4 and 5, three independently analyzed microsphere‐based statistical units were included for each condition. ImageJ software tracked every particle without additional processing, allowing free, nonspecifically attached, and specifically bound AuNPs to be distinguished. Only specifically bound particles were counted; the resulting density per unit area provided the quantitative readout.

## Author Contributions

H.Y., T.Z. and P.Z. developed the concept. T.Z. performed the experiments with assistance from L.L. and G.G. H.Y., H.Z. and S.L. supervised the project. All authors were involved in preparing the figures and the original draft. H.Y. reviewed and edited. All authors discussed the results. L.L. and G.G. contributed equally to this work.

## Funding

This work was supported by the National Natural Science Foundation of China (NSFC, Nos. 62175252, 62205366, and 82227806), Guangdong Basic and Applied Basic Research Foundation (2024A1515012320), Shenzhen Science and Technology Innovations Committee (JSGGZD20220822095200001), and Shenzhen Medical Academy of Research and Translation (E250200411).

## Conflicts of Interest

The authors declare no conflicts of interest.

## Supporting information




**Supporting File 1**: advs76076‐sup‐0001‐SuppMat.docx.

**Supporting File 2**: advs76076‐sup‐0002‐VideoS1.mp4.

**Supporting File 3**: advs76076‐sup‐0003‐VideoS2.mp4

## Data Availability

The data that support the findings of this study are available from the corresponding author upon reasonable request.
