# Supplementary material for: Dynamic Single‐Binding Event Profiling With on‐Chip Microlenses for Wash‐Free Digital Biosensing
Source: Adv Sci (Weinh). 2026 Jun 28;13(46):e76076. doi: 10.1002/advs.76076 (PMC13337108; doi:10.1002/advs.76076)
Supplement: Supplementary file 1 [file ADVS-13-e76076-s002.docx]

Supporting Information

Dynamic single-binding event profiling with on-chip microlenses for wash-free digital biosensing

*Tingting Zhan, Lianyu Lu, Guoqiang Gu, Pengcheng Zhang, Jienan Shen, Xiaotian Tan, Wei Ye, Shi Hu, Yi Zhang, Hao Yu, Shaoqin Liu*, Hairong Zheng*, Hui Yang**

**Table of contents**

1. Quantification of net intensity and SNR of individual AuNPs;
2. Quantitative robustness analysis of the kinetic-based decision logic;
3. Effect of attached-particle exclusion on calibration performance;
4. Reproducibility of kinetic classification across concentrations and matrices;
5. Fig. S1. Characterization of as-prepared AuNP-labeled antibody;
6. Fig. S2. Fabrication of the microlens chip;
7. Fig. S3. Characterization of the microlens chip;
8. Fig. S4. Schematic illustration of the modification of capture antibody on chip surface;
9. Fig. S5. Correlation between the measured intensity and the diameter of single AuNPs;
10. Fig. S6. Correlation between the measured intensity of AuNPs and the diameter of microlens;
11. Fig. S7. AuNPs on a microlens chip imaged with different objective lenses;
12. Fig. S8. Robustness analysis of the attached-particle exclusion window;
13. Fig. S9. Structure diagram of self-developed portable optical device;
14. Fig. S10. Control experiments for cTnI detection;
15. Fig. S11. Replicate-based total count statistics for cTnI detection;
16. Fig. S12. Count-based SNR analysis before and after attached-particle exclusion in PBS and undiluted serum;
17. Fig. S13. Reproducibility of digital counting before and after attached-particle exclusion;
18. Fig. S14. Effect of attached-particle exclusion on calibration performance in PBS and undiluted serum.
19. Videos S1 and S2. Dynamic binding processes of individual AuNPs over time.
20. Tables S1, S2 and S3.
21. References

**Quantification of net intensity and SNR of individual AuNPs**

To quantitatively evaluate the imaging performance of individual AuNPs, the net intensity and signal-to-noise ratio (SNR) were calculated from bright-field images using a local region-of-interest (ROI)-based analysis. For each AuNP, a circular ROI centered on the particle was selected as the signal region, and a surrounding local background region was used to estimate the background level and its fluctuation.

The net intensity (I_net_) of an individual AuNP was defined as the difference between the mean intensity of the particle ROI and the mean intensity of the local background region:

$$I_{net}= \overline{I}_{sig}- \overline{I}_{bg}$$

where $\overline{I}_{sig}$ is the mean intensity within the particle ROI and $\overline{I}_{bg}$ is the mean intensity of the local background region.

The signal-to-noise ratio (SNR) was calculated as:

$$SNR= \frac{I_{net}}{\sigma_{bg}}$$

where $\sigma_{bg}$ is the standard deviation of the intensity in the local background region.

All calculations were performed using the same ROI definition and analysis procedure for images acquired with and without the microlens chip under identical optical conditions.

**Quantitative robustness analysis of the kinetic-based decision logic**

To quantitatively assess the robustness of the kinetic-based decision logic, we varied the attached-particle exclusion window from 0 to 10 s and evaluated the resulting blank counts, low-concentration positive counts, and count-based SNR (SNR_count_). The analysis focused on attached particles because free particles are transient and typically appear in only a single frame, allowing them to be effectively excluded during trajectory tracking. In contrast, initially attached particles persist in the field of view and therefore constitute the major potential source of false-positive detection counts.

As shown in Fig. S8, the blank count was high when no exclusion window was applied, whereas increasing the window to 2 s substantially reduced the blank background. The low-concentration (5 pg/mL) positive count decreased relative to the unexcluded condition but reached a plateau after the 2 s window, indicating that most nonspecific initially attached particles had been removed while the target-associated signal was largely retained. Extending the window to 4, 6, or 10 s produced only marginal further reductions in blank counts but gradually diminished the positive counts, suggesting that overly stringent exclusion may begin to remove true detection events. Consistently, the SNR_count_ increased after application of the 2 s window and remained high across the tested threshold range.

We further performed a reference-threshold-based ROC-like analysis, taking the net count obtained with the optimized 2 s window as the reference positive count (Fig. S8b). This analysis serves as a threshold-sensitivity assessment rather than an independent diagnostic ROC analysis. The resulting curve exhibited a high AUC, supporting the conclusion that the 2 s exclusion window provides a favorable balance between false-positive suppression and signal retention. Together, these results demonstrate that the classification rule is robust to moderate threshold variations and that potential misclassification has limited impact on the reported digital counting accuracy and analytical performance.

The count-based SNR (SNR_count_) was defined as:

$${SNR}_{count}= \frac{\overline{N}_{sig}- \overline{N}_{blk}}{\sigma_{blk}}$$

where $\overline{N}_{sig}$ and $\overline{N}_{blk}$ are the mean signal counts for target analyte and blank, respectively, and $\sigma_{blk}$ is the standard deviation of the blank counts.

**Effect of attached-particle exclusion on calibration** **performance**

To evaluate how retained attached particles affect counting uncertainty and analytical performance, we compared calibration curves before and after attached-particle exclusion in PBS and undiluted serum (Fig. S14) and compiled the corresponding blank statistics and LOD values in Table S3. Note that the “before attached-particle exclusion” data do not represent completely unprocessed total particle counts. Single-frame free particles were already removed during trajectory construction because they appear transiently and do not form persistent tracks over the 2 s acquisition interval. Therefore, this comparison primarily isolates the contribution of initially attached particles to false-positive background and counting uncertainty.

In PBS, including initially attached particles elevated the signal counts and degraded the calibration fit. After exclusion, the fit improved (R^2^ from 0.91 to 0.99), the linear dynamic range expanded by one order of magnitude, and the corresponding LOD improved from 1.32 pg/mL to 0.051 pg/mL. These changes indicate that attached particles can introduce concentration-independent false-positive background and increase counting uncertainty if not excluded. Consequently, attached-particle exclusion is important for achieving accurate counting and reliable calibration in PBS. In undiluted serum, the calibration curves before and after exclusion were nearly identical, both showing good linearity (R^2^ ≈ 0.99), and the LOD values were comparable. These results suggest that initially attached particles contribute minimally to counting uncertainty in serum, consistent with the low number of particles detected during the initial exclusion window. The remaining serum-related uncertainty is therefore more likely attributed to matrix-dependent slow background accumulation and mass-transport limitations rather than early attached-particle misclassification.

Together, these results demonstrate that the influence of attached-particle-derived misclassification is matrix-dependent. The kinetic classification rule effectively suppresses attached-particle-derived false-positive counts in PBS without distorting the calibration response in serum, confirming the robustness of the digital counting strategy across different sample matrices.

**Reproducibility of kinetic classification across concentrations and matrices**

To evaluate the reproducibility of the kinetic classification rule across different analyte concentrations and sample matrices, we calculated the coefficient of variation (CV) of digital counts before and after attached-particle exclusion in PBS and undiluted serum (Fig. S13). In PBS, the CV after exclusion remained low across the entire concentration range, demonstrating stable counting following removal of initially attached background particles and confirming that the classification rule does not introduce substantial counting variability. In undiluted serum, the CV was higher at the lowest concentrations, as expected given the smaller number of target-associated events and greater matrix-related fluctuation, but decreased monotonically as the cTnI concentration increased, indicating improved counting stability at higher event throughput. Moreover, the similar CV trends before and after attached-particle exclusion in serum indicate that initially attached particles are not the dominant source of counting uncertainty in this matrix. Collectively, these results affirm the reproducibility of the kinetic classification rule across analyte concentrations and serum backgrounds.

Fig. S1. UV–vis absorption spectrum of the as-prepared AuNP-labeled antibody. The UV–vis absorption spectrum exhibited maxima at 280 nm (protein) and 550 nm (gold surface plasmon), confirming successful preparation of AuNP-labeled antibody.

Fig. S2. Fabrication workflow of the microlens chip. Barium-titanate-glass (BTG) microspheres were embedded in a pre-spin-coated polydimethylsiloxane (PDMS) layer on a glass substrate.

Fig. S3. Structural and optical characterization of the microlens chip. (a). Photograph of the chip; each chip contains five detection zones. (b). SEM overview of the microlens array. (c). SEM of AuNPs immobilized on a microlens; inset: magnified view of individual AuNPs. (d). Bright-field micrograph of the microlens array. (e). The effective imaging region of a single microlens (white dashed circle). (f). Bright-field image of discrete AuNPs within one effective microlens-assisted imaging region.

Fig. S4. Surface chemistry for immobilizing capture antibody (cAb). APTES = (3-aminopropyl) triethoxysilane, GA = glutaraldehyde, BSA = bovine serum albumin.

Fig. S5. Integrated intensity versus diameter for single AuNPs. The detection limit was 60 nm.

Fig. S6. Integrated intensity of AuNP spots as a function of microlens diameter.

Fig. S7. Imaging performance across objective magnifications. (a). Bright-field images of AuNPs on a microlens chip acquired with 10×, 20×, and 40× objectives. Individual AuNPs appear as point-spread-function-defined spots. As long as they are not in close proximity, all AuNPs visible under a 40× objective lens are also discernible under 10× and 20× objective lenses. (b). Extracted particle positions (red spots) from (a); pairs 1-3 illustrate (1) resolved, (2) marginally resolved, and (3) unresolved AuNPs under a 10× objective. (c). Scatter plot comparing counts obtained with 20× and 40× objectives.

Fig. S8. Robustness analysis of the attached-particle exclusion window. (a). Signal counts of blank and 5 pg/mL cTnI samples, together with count-based SNR, under different attached-particle exclusion windows of 0, 2, 4, 6, and 10 s. (b). Reference-threshold-based ROC-like analysis of the attached-particle exclusion window. The net count obtained at the optimized 2 s threshold was used as the reference positive count.

Fig. S9. Illustration of self-developed portable optical device. (a). Structure diagram of self-developed portable optical device for signal read-out. (b). The photograph of the self-developed portable optical device.

Fig. S10. Control experiments for cTnI detection. Real-time digital counts under four conditions: (a). 5 pg/mL cTnI; (b). no cTnI; (c). no capture antibody; (d). neither capture antibody nor cTnI.

Fig. S11. Replicate-based total count statistics for cTnI detection. (a). Total digital signal counts at different cTnI concentrations in PBS after 19 min of tracking. (b). Total digital signal counts at different cTnI concentrations in undiluted serum after 30 min of tracking. Counts were obtained from three replicate microsphere-based statistical units at each concentration.

Fig. S12. Count-based SNR analysis before and after attached-particle exclusion. Signal counts for blank and 5 pg/mL cTnI in PBS and undiluted serum, compared before and after attached-particle exclusion. “Before” and “After” denote the conditions before and after the exclusion step.

Fig. S13. Reproducibility of digital counting before and after attached-particle exclusion. Coefficient of variation (CV) of digital signal counts as a function of cTnI concentration, comparing results before and after attached-particle exclusion in (a) PBS and (b) undiluted serum.

Fig. S14. Effect of attached-particle exclusion on calibration performance. (a). Calibration curves in PBS before and after attached-particle exclusion. (b). Calibration curves in undiluted serum before and after attached-particle exclusion. Single-frame free particles were excluded during trajectory construction; therefore, the “before” counts primarily represent detection particles and initially attached particles.

Video S1. Real-time binding events at 0 pg/mL cTnI; playback is 10× real time.

Video S2. Real-time binding events at 5 pg/mL cTnI; playback is 10× real time.

**Table S1.** Comparison of the proposed single-binding event counting method with other commercial technologies for cTnI detection

Estimated instrument cost (EC): $ = EC< 20000 USD); $$ = (20000 USD < EC< 50000 USD); $$$= (EC > 50000 USD);

Assay duration (AD): ▼= (AD < 2 steps); ▼▼= (AD > 2 steps);

| Method | Conventional ELISA | Quanterix SiMoA HD-I | UniCel DxI 800 (Beckman Coulter) | SMC × PRO^TM^ (MilliporeSigma) | Proposed method |
| --- | --- | --- | --- | --- | --- |
| Sample volume (μL) | 50-100 | 42 | 55 | 100 | 10 |
| LOD (pg/mL) | 4.4 | 0.010 | 2 | 0.11 | 0.051 |
| LDR (pg/mL) | 31.3-4000 | > 4 logs | 30.7-27027 | > 4 logs | 5-10000 |
| Estimated instrument cost | $$ | $$$ | $$$ | $$$ | $ |
| Assay time | 1.5 h | 45 min | > 30 min | 32 min | 29 min |
| Wash-free | No | No | No | No | Yes |
| Assay duration | ▼▼ | ▼▼ | ▼▼ | ▼▼ | ▼ |
| Ref | a | [1] | b | [2] | this work |

Note: LDR: linear dynamic range; LOD: limit of detection; Assay time: total sample-to-result time, defined as the overall time from sample mixing to result readout, encompassing the pre-incubation step and the subsequent on-chip counting/analysis time. The proposed method uses a 10-min pre-incubation followed by on-chip counting (19 min for PBS, 30 min for serum), for a total sample-to-result time of 29 min under the PBS protocol.

a: https://www.abcam.com/products/elisa/human-cardiac-troponin-i-elisa-kit-ab200016.html

b: https://www.beckmancoulter.com/en/products/immunoassay/dxi-800

**Table S2.** Comparison of the proposed single-binding event counting method with other previously published digital immunoassays

| Scheme | Ref | Sample volume (μL) | LDR (pg/mL) | LOD (pg/mL) | Operation steps | Assay time (min) | Objective parameters | Probe size |
| --- | --- | --- | --- | --- | --- | --- | --- | --- |
| Fluorescent imaging | [3] | 20 | 0−50000 | 170 | 5 | > 2h | 20×/ 0.4 NA | Not available |
| Differential bright-field imaging | [4] | 10 | 5-5000 | 1.4 | 1 | 10 min | 40×/ 0.75 NA | 60 nm |
| Plasmonic imaging | [5] | 100 | 4−12500 | 2.8 | 5 | 25 min | 60×/ 1.49 NA | 150 nm |
| Fluorescent imaging | [6] | 10 | 3 logs | 0.093 | 2 | >1 h | 40× (NA: Not available) | 550 nm |
| Bright-field imaging | [7] | 50 | 3.9-6250 | 8.4 | 6 | 14 min | 40×/ 0.75 NA | 150 nm |
| Scattered-light imaging | this work | 10 | 5-10000 | 0.051 | 1 | 29 min | 40×/ 0.55 NA | 80 nm |

Note: LDR: linear dynamic range; LOD: limit of detection; NA: numerical aperture. Assay time: total sample-to-result time, defined as the overall time from sample mixing to result readout, encompassing the pre-incubation step and the subsequent on-chip counting/analysis time. The proposed method uses a 10-min pre-incubation followed by on-chip counting (19 min for PBS, 30 min for serum), for a total sample-to-result time of 29 min under the PBS protocol and 40 min for serum.

**Table S3.** Blank statistics, fitted equations, and LOD values before and after attached-particle exclusion in PBS and undiluted serum

| Matrix | Condition | Blank mean | Blank SD | Fitted equation | LOD (pg/mL) |
| --- | --- | --- | --- | --- | --- |
| PBS | Before attached exclusion | 14.00 | 5.20 | y=24.83+39.64 lg(x) | 1.32 |
| PBS | After attached exclusion | 2.00 | 1.73 | y=30.38+17.90 lg(x) | 0.051 |
| Serum | Before attached exclusion | 5.67 | 1.15 | y=6.49+10.45 lg(x) | 1.79 |
| Serum | After attached exclusion | 5.33 | 1.52 | y=6.00+10.04 lg(x) | 2.44 |

**References**

1. D. H. Wilson, D. M. Rissin, C. W. Kan, et al., “The Simoa HD-1 analyzer: A novel fully automated digital immunoassay analyzer with single-molecule sensitivity and multiplexing,” *SLAS Technology* 21 (2016): 533–547, https://doi.org/10.1177/2211068215589580.

2. J. Hwang, M. Banerjee, A. S. Venable, et al., “Quantitation of low abundant soluble biomarkers using high sensitivity Single Molecule Counting technology,” *Methods* 158 (2019): 69–76, https://doi.org/10.1016/j.ymeth.2018.10.018.

3. Q. Zhang, J. Li, X. Pan, et al., “Low-numerical aperture microscope objective boosted by liquid-immersed dielectric microspheres for quantum dot-based digital immunoassays,” *Analytical Chemistry* 93 (2021): 12848–12853, https://doi.org/10.1021/acs.analchem.1c02709.

4. Y. Wang, Y. Yang, C. Chen, et al., “One-step digital immunoassay for rapid and sensitive detection of cardiac troponin I,” *ACS Sensors* 5 (2020): 1126–1131, https://doi.org/10.1021/acssensors.0c00064.

5. W. Jing, Y. Wang, Y. Yang, et al., “Time-resolved digital immunoassay for rapid and sensitive quantitation of procalcitonin with plasmonic imaging,” *ACS Nano* 13 (2019): 8609–8617, https://doi.org/10.1021/acsnano.9b02771.

6. K. Akama, N. Iwanaga, K. Yamawaki, et al., “Wash- and amplification-free digital immunoassay based on single-particle motion analysis,” *ACS Nano* 13 (2019): 13116–13126, https://doi.org/10.1021/acsnano.9b05917.

7. Y. Wang, W. Jing, N. Tao, et al., “Probing single-molecule binding event by the dynamic counting and mapping of individual nanoparticles,” *ACS Sensors* 6 (2021): 523–529, https://doi.org/10.1021/acssensors.0c02184.
